# Psoriasis and Psoriatic Arthritis Cardiovascular Disease Endotypes Identified by Red Blood Cell Distribution Width and Mean Platelet Volume

**DOI:** 10.3390/jcm9010186

**Published:** 2020-01-09

**Authors:** Rosalynn RZ Conic, Giovanni Damiani, Kory P. Schrom, Amy E. Ramser, Chunlei Zheng, Rong Xu, Thomas S. McCormick, Kevin D. Cooper

**Affiliations:** 1 Department of Dermatology, Case Western Reserve University, Cleveland, OH 44106, USA; ruzica.conic@gmail.com (R.R.C.); dr.giovanni.damiani@gmail.com (G.D.); tsm4@case.edu (T.S.M.); 2 Department of Dermatology, University Hospitals, Cleveland Medical Center, Cleveland, OH 44106, USA; kory.schrom@gmail.com (K.P.S.); amy.ramser@uhhospitals.org (A.E.R.); 3 Clinical Dermatology, IRCCS Istituto Ortopedico Galeazzi, 20161 Milan, Italy; 4 Department of Biomedical, Surgical and Dental Sciences, University of Milan, 20161 Milan, Italy; 5 Department of Population and Quantitative Health Sciences, Case Western Reserve University, Cleveland, OH 44106, USA; zhengcl75@gmail.com (C.Z.); rxx@case.edu (R.X.); 6 Department of Dermatology, Veteran’s Administration Medical Center, Cleveland, OH 44106, USA

**Keywords:** Psoriasis, psoriatic arthritis, MACE, red cell distribution width (RDW), mean platelet volume (MPV), endotypes, precision medicine

## Abstract

In a subset of psoriasis (PsO) and psoriatic arthritis (PsA) patients, the skin and/or joint lesions appear to generate biologically significant systemic inflammation. Red cell distribution width (RDW) and mean platelet volume (MPV) are readily available clinical tests that reflect responses of the bone marrow and/or plasma thrombogenicity (e.g., inflammation), and can be markers for major adverse cardiac events (MACE). We aimed to evaluate if RDW and MPV may be employed as inexpensive, routinely obtained biomarkers in predicting myocardial infarction (MI), atrial fibrillation (AF), and chronic heart failure (CHF) in psoriatic and psoriatic arthritis patients. The study was divided into two parts: (a) case control study employing big data (Explorys) to assess MPV and RDW in psoriasis, psoriatic arthritis and control cohorts; (b) a clinical observational study to validate the predictive value of RDW and to evaluate RDW response to anti-psoriatic therapies. We used Explorys, an aggregate electronic database, to identify psoriatic patients with available MPV and RDW data and compared them to gender and age matched controls. The incidence of myocardial infarction (MI), atrial fibrillation (AF), and chronic heart failure (CHF) was highest among patients with both elevated RDW and MPV, followed by patients with high RDW and normal MPV. RDW elevation among PsA patients was associated with an increased risk of MI, AF, and CHF. In a local clinical cohort, high RDWs were concentrated in a subset of patients who also had elevated circulating resistin levels. Among a small subset of participants who were treated with various systemic and biologic therapies, and observed over a year, and in whom RDW was elevated at baseline, a sustained response to therapy was associated with a decrease in RDW. RDW and MPV, tests commonly contained within routine complete blood count (CBC), may be a cost-effective manner to identify PsO and PsA patients at increased risk of MACE.

## 1. Introduction

Psoriasis is a chronic systemic inflammatory disease affecting approximately 2–3% of the population. Due to the chronicity and distal effects of inflammation associated with psoriasis, numerous comorbidities have been identified in affected patients [1,2]. One major concern is the reported elevated risk of major adverse cardiac events (MACE) in patients with psoriasis [1,3,4,5]; however, one concern is the differential impact of psoriatic patients undergoing different therapies, that makes estimation of psoriasis related CVD risk challenging. Using imaging, our group previously identified an increased prevalence of undiagnosed atherosclerosis among psoriasis patients under 40 years of age, which predisposes to MACE [6]. Other associated cardiovascular conditions such as hypertension and renal disease are also increased [6,7]. However, it is unlikely that each and every psoriasis patient has to be managed for increased cardiovascular risk, so there is a pressing need to enable dermatologists to more precisely identify and counsel the subset of psoriasis patients, also referred to as an endotype, who do have significant risk for developing accelerated cardiovascular disease (CVD). This latter group is the subset who experience effects of their psoriasis on distant organs such as the systemic vasculature or bone marrow, which enables a mechanistic opportunity to identify those psoriasis patients with endotypic markers of an extreme phenotype (i.e., increased MACE risk). 

The literature in cardiovascular research is rich with complicated, expensive measurements to assess potential risk for cardiovascular complications [8]. Whereas these measurements are appreciated for their elegance, instituting them into routine dermatologic clinical practice is impractical. Therefore, identification of simple, reliable markers which would identify psoriasis patients who should be more closely monitored for developing MACE would be of immediate clinical benefit. Upon review of the cardiovascular literature, two potential markers of increased CVD risk that are available within a standard complete blood count (CBC) were identified -red cell distribution width (RDW) and mean platelet volume (MPV) [9,10]. RDW is a measurement of the variability in size and volume of circulating red blood cells, and is employed routinely in CBCs in the differential diagnosis of anemia. MPV is a measurement of the average size of platelets found in circulation, which is generally reflective of platelet activation, and is also typically reported in complete blood count (CBC) measurements. Recently, these markers have been associated with increased MACE in large undifferentiated patient populations and in HIV patient cohorts [8,11,12,13]. Limited evidence for elevated levels of MPV and RDW in a greater percentage of psoriasis patients has been identified previously in small sample size cohorts; however, the risk of MACE and CVD in psoriasis patients with aberrant RDW and MPV values remains to be determined [14,15,16]. 

Recently, we reviewed current evidence in psoriasis suggesting that cutaneous inflammation can result in bone marrow dysbiosis [17]. Specifically, psoriasis patients have a disordered bone marrow cytokine environment, affecting both inflammatory mediators and growth factors [18,19,20]. Therefore, we hypothesize that a subset of psoriasis patients have sufficient magnitude, duration, and/or quality of inflammation to exert distal effects on the bone marrow production of RBCs and platelets resulting in their disordered release or functionality in the circulation that is detectable by simple CBC testing for RDW and MPV. Prior psoriasis studies on markers of local inflammation which are associated with an increased risk of CVD include myeloperoxidase, a pro-inflammatory heme protein released by myeloid cells (most likely released into circulation by MPO-producing skin-infiltrating myeloid cells), and resistin, an adipose-tissue derived hormone (most likely from adipose tissue subjacent to psoriasis plaques) [21,22,23,24,25]. In addition, we and others demonstrated that myeloid cells are altered in psoriasis patients, where the number of monocyte-derived suppressor cells (MDSCs) and intermediate (CD14^+^CD16^+^) monocytes are increased in psoriasis patients, potentially as a result of similarly disordered myeloid cell release [24,26,27,28,29,30,31,32]. 

The goal of the present study was to assess whatever or not elevated MPV and RDW values identify subsets of psoriasis or psoriatic arthritis patients with a higher risk to develop cardiovascular diseases.

## 2. Experimental Section

### 2.1. Study Design

The study was performed in two parts: (1) a case-control study using Explorys data and (2) data using University Hospitals Cleveland Medical Center psoriasis patient cohorts. The Explorys-based study was considered non-human subject research by the Institutional Review Board (IRB). For the cohorts derived from the University Hospitals Cleveland Medical Center Data, the IRB numbers are 04-13-21 and 05-95-03.

### 2.2. Explorys

Explorys is an aggregate electronic health record database containing over 50 million patient records compiled from 26 different healthcare systems. Data is pulled from the electronic health record of these resources daily. Explorys uses Systematized Nomenclature of Medicine Clinical Terms (SNOMED-CT) coding for diagnosis and Logical Observation Identifiers Names and Codes (LOINC) for lab test result measurements. Counts of patients with psoriasis were rounded to the nearest 10, and counts with less than 10 patients available are equivalent to 0; however our results due to the size of the cohort, were unaffected. The University Hospitals Cleveland Medical Center Institutional Review Board deemed studies using Explorys as the data set of record as exempt because all data is de-identified.

Patients aged 18–65 years with SNOMED-CT codes for psoriasis and available RDW measurements and/or available MPV measurements were selected from Explorys (Run date 07/09/2018) (Supplementary Figure S1). Patients with associated SNOMED-CT codes related to psoriasis types different from plaque psoriasis, namely “Impetigo herpetiformis”, “Pustular psoriasis”, and “Guttate psoriasis”, and related to diseases known to increase MACE, namely “Diabetes mellitus” [33], “Crohn’s disease” [34], “Rheumatoid arthritis” [35], “Generalized atherosclerosis” [36] were excluded from the cohort. Hematological disorders were not used as exclusion criteria in this study because in the CHARM program and in the Duke Databank RDW was not related to hemoglobin levels [37]. This cohort was then separated into 3 groups: controls, psoriasis (PsO) patients without psoriatic arthritis, and patients with both psoriasis and psoriatic arthritis (PsA) patients. 

Because in the reported literature of a large cohort study, RDW, after adjusting for hemoglobin levels, was described as an independent predictor of both morbidity and mortality for MACE-related events, we decided not to exclude anemic patients.

**Figure 1:**
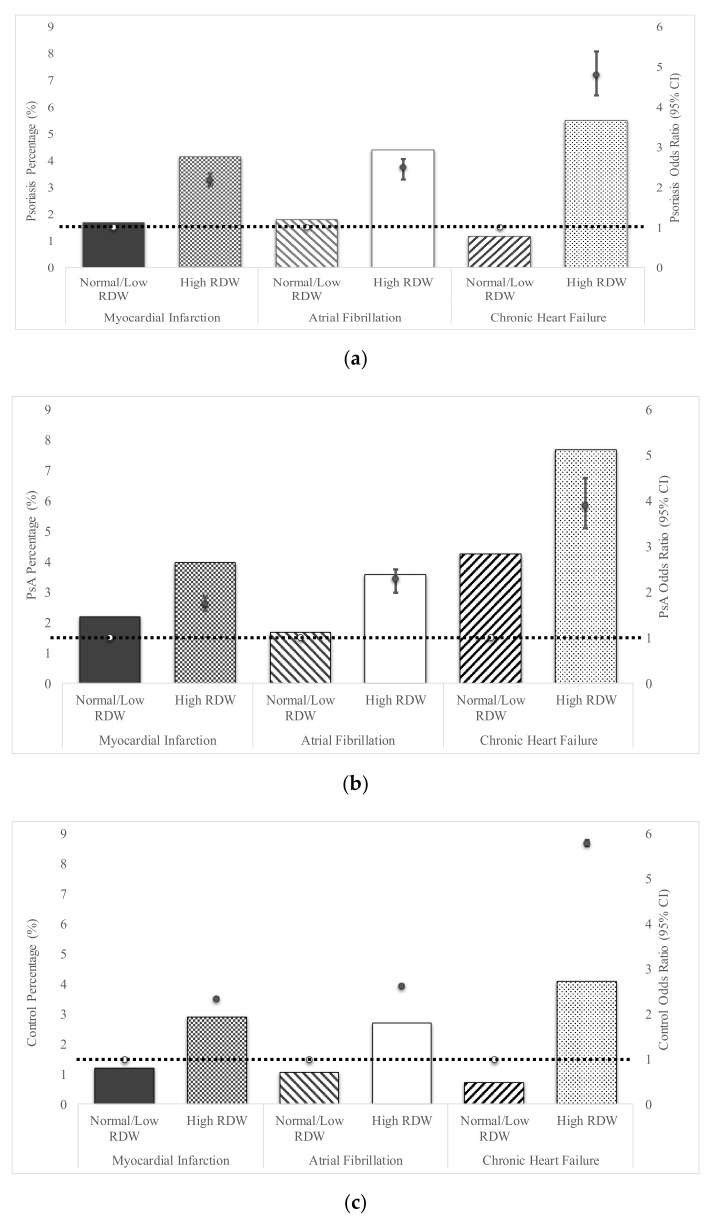
Development of Cardiovascular Disease based on high versus normal red cell distribution width (RDW) for Psoriasis patients, Psoriatic Arthritis patients, and Controls. The frequency and odds ratio of presenting with three distinct types of cardiovascular diseases based upon RDW status (high versus normal/low, as defined by Explorys) is shown for each group. The scale for the percentage of patients (PsO, PsA) or controls exhibiting each combination is demarcated on the left hand side of the graph, while the odds ratios (OR) scale is listed on the right hand side. Odds Ratios were adjusted for age, gender and hypertension. Open circles connected by the dashed line represent the reference value (1) for each OR. Odds Ratios are represented by solid circles and error bars, which represent 95% confidence intervals. The percentage of psoriasis patients (**a**), psoriatic arthritis patients (**b**) or control subjects (**c**) exhibiting either myocardial infarction (MI), arial fibrillation (AF) and chronic heart failure in psoriasis patients based on RDW expression levels is shown. Prediction of odds ratio for development of the three types of heart failure in psoriasis (**a**) psoriatic arthritis (**b**) and controls (**c**) are quantified on the right axis.

PsA patients were identified using the SNOMED-CT terms “psoriasis with arthropathy”, “psoriatic arthritis with spine involvement”, or “psoriatic arthritis with distal interphalangeal joint involvement”. PsO patients were identified by excluding those with PsA according to the above SNOMED-CT codes. Interestingly, the use of ICD coding for PsO and PsA has been validated to slightly underestimate the diagnosis of PsA [38]. Control patients were identified by excluding patients with either PsO or PsA, in addition to the terms excluded above (Figure 1). 

Explorys assigns categorical classification of RDW and MPV values into three groups (high, normal and low): RDW was labeled as high (>14.5%), normal (11.5–14.5%) or low (<11.5%) levels, while MPV was labeled high (>12.3 fL), normal (9.4–12.3 fL) and low (<9.4 fL). RDW was considered elevated if the subject has a single record that exceeded the normal range. Despite the heterogeneity existing in normality ranges of both RDW and MPV, each lab uploads their values and they were normalized using the ranges reported previously in order to enable data comparison. Explorys does not distinguish between ambulatory and inpatient values. Cardiac events selected for analysis were myocardial infarction (MI), atrial fibrillation (AF), and chronic heart failure (CHF).

The chosen covariates were age, gender, and hypertension in agreement with Ye and colleagues [39].

Smoking status was determined through a self-reported field in Explorys. A smoker was considered to be anyone who indicated that they were a smoking at any time during the study period.

### 2.3. University Hospitals Cleveland Medical Center Data

In order to further account for possible RDW confounders impossible to address within Explorys, we examined RDW in a cohort of psoriatic patients where we manually excluded patients who were binge drinkers and vegans or vegetarians, smokers with recent (<5 years) activity, hematological malignancies or drug intake that could modify bone marrow function. We also mimicked the Explorys criteria excluding individuals age <21, diabetes, body mass index (BMI) >26, and metabolic syndrome. 

To assess how RDW responds to distal organ inflammation as well as response to therapy, we analyzed data obtained from psoriasis patients treated clinically at the Murdough Family Center for Psoriasis with available RDW, resistin, and myeloperoxidase (MPO) values (*n* = 75). A sub-cohort of patients were followed in an observational study for one year with repetitive CBC’s (*n* = 24). These patients received standard-of-care systemic agents, and therapy was changed if patients did not respond after 3 months. Plasma, serum, and cellular biomarkers were collected at baseline and every 3 months. Among the 24 patients, 4 patients had baseline high RDW values and were analyzed for effects of medication on RDW levels. Psoriasis severity was determined by the psoriasis area and severity index (PASI) [40].

### 2.4. Statistical Analysis

The relationship between RDW, resistin, and MPO was assessed using chi-square test. Spearman correlation was used to assess the correlation between RDW and PASI. R statistical software (version 3.4.1) was used to perform all analyses [41]. Chi-squared tests were used to compare the difference of categories or subcategories of two cohorts. Odds ratios were constructed using logistic regression. Adjustments were made for age, gender, and hypertension. A sensitivity analysis for anemia was performed and demonstrated that results did not differ significantly based on this criteria.

## 3. Results

### 3.1. Demographics

After exclusion criteria were applied, Explorys contained 10,747,320 records of subjects with RDW or MPV recorded. Of these subjects, 91,190 had PsO and 22,220 of these also exhibited PsA (Table 1). 

Controls were slightly younger, compared to patients with either PsO or PsA. The majority of patients across all groups were Caucasian. Obesity was most common among PsA patients followed by controls. Hypertension (combining Stage 1 and Stage 2) was present in 38% of controls, 59% of PsO patients, and 53% of PsA patients, which is concordant with other population data. Elevated RDW was present in 5210 (23.45%) PsA patients, 19,480 (21.36%) PsO patients, and 1,943,130 (18.27%) controls. Elevated MPV was found in 1850 (8.33%) PsA patients, 7900 (8.66%) PsO patients, and 747,140 (7.03%) controls.

### 3.2. Erythrocyte Distribution Width and Cardiovascular Disease

After adjusting for age, gender, and hypertension, elevated RDW among psoriasis patients demonstrated significantly increased risk for MI (OR 2.18, 95%CI 2.02–2.35), AF (OR 2.5, 95%CI 2.2–2.7), and CHF (OR 4.8 95%CI 4.3–5.4) relative to psoriasis patients with normal/low RDW (Figure 1a). Those with PsA and elevated RDW were also at increased risk for MI (OR 1.75, 95%CI 1.51–2.02), AF (OR 2.3, 95%CI 1.9–2.8), and CHF (OR 3.9 95%CI 2.5–4.3) relative to PsA patients with normal/low RDW (Figure 1b). Validating prior studies, control patients without PsO or PsA with elevated RDW had an increased risk of MI infarction (OR 2.34, 95%CI 2.31–2.37), AF (OR 2.62, 95%CI 2.59–2.65), and CHF (OR 5.8 95%CI 5.74–5.87) compared to control patients with normal/low RDW (Figure 1c).

In comparison to control subjects with high RDW, patients with PsO or PsA and high RDW had even further increased risk of myocardial infarction (PsO OR 1.45, 95%CI 1.32–1.59; PsA OR 1.39, 95% CI 1.28–1.55), atrial fibrillation (PsO OR 1.36, 95%CI 1.25–1.47; PsA OR 1.52, 95%CI 1.43–1.57), and heart failure (PsO OR 1.36, 95%CI 1.25–1.47; PsA OR 1.52, 95%CI 1.43–1.57) (Supplementary Figure S2).

### 3.3. Mean Platelet Volume and Cardiovascular Disease

**Figure 2:**
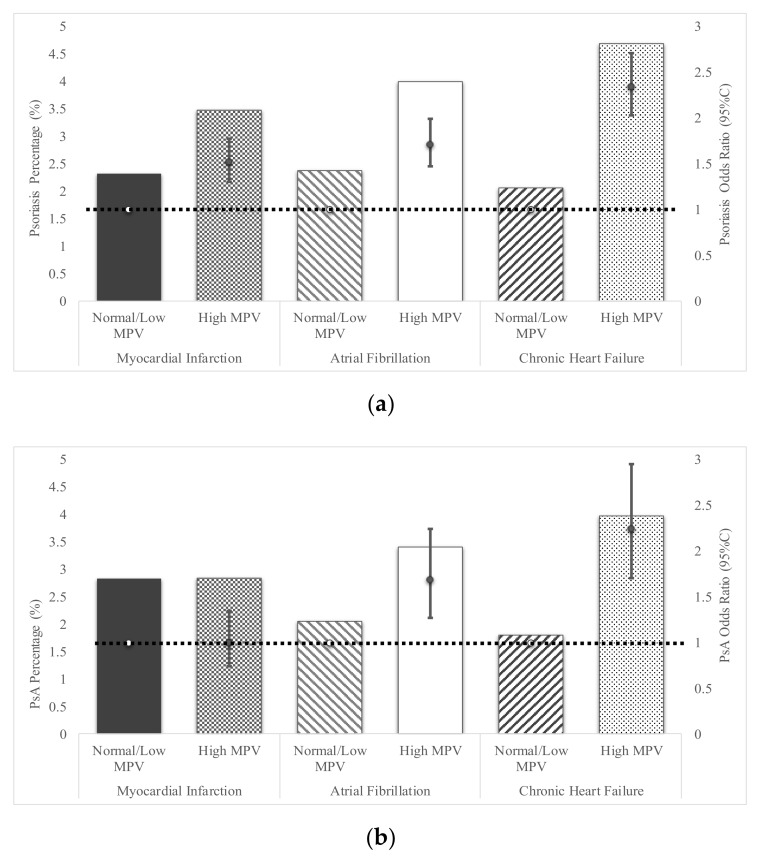

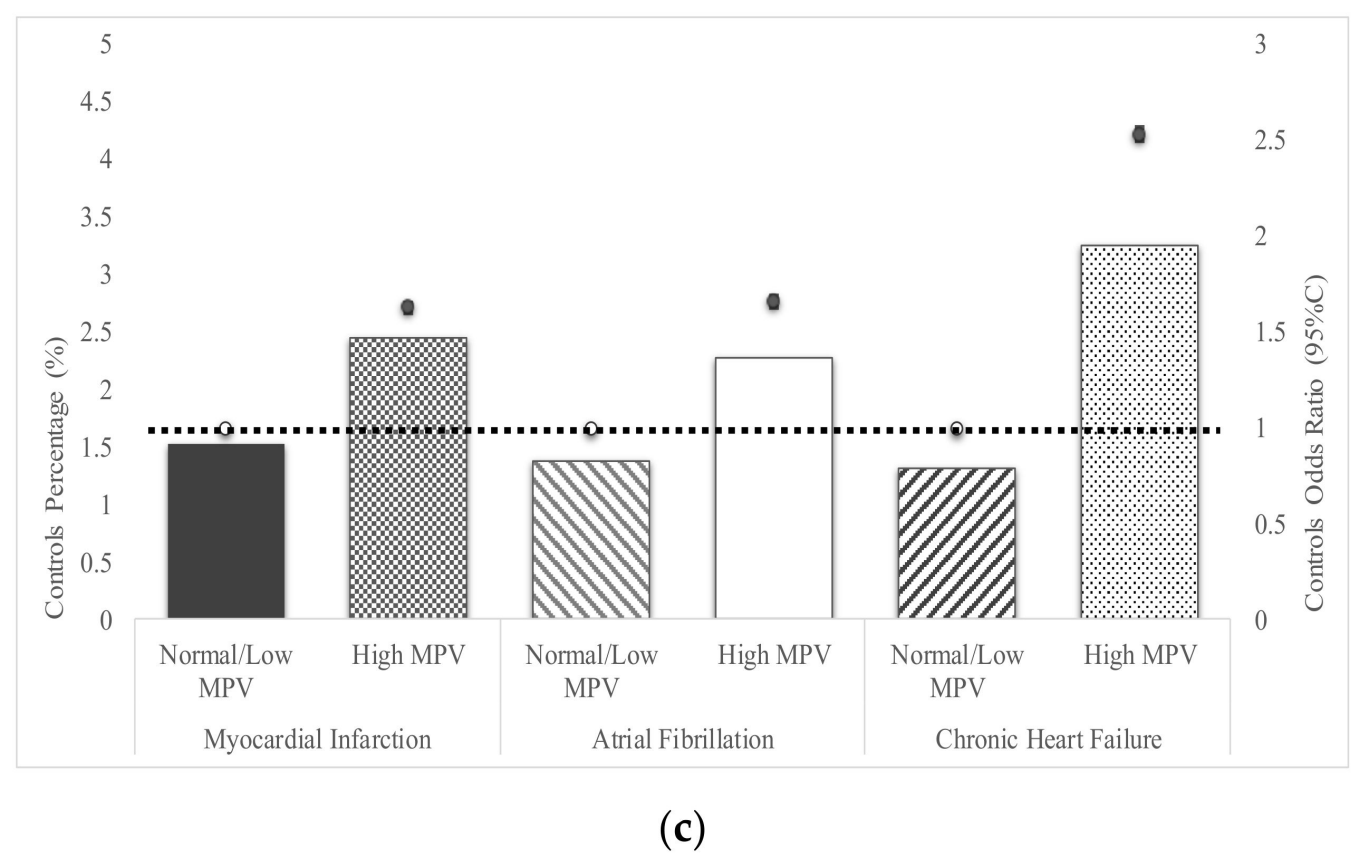
Development of Cardiovascular Disease based on high versus normal mean platelet volume (MPV) for Psoriasis patients, Psoriatic Arthritis patients, and Controls. The frequency and odds ratio of presenting with three distinct types of cardiovascular diseases based upon RDW status (high versus normal/low, as defined by Explorys) is shown for each group. The scale for the percentage of patients (PsO, PsA) or controls exhibiting each combination is demarcated on the left hand side of the graph, while the odds ratios (OR) scale is listed on the right hand side. Odds Ratios were adjusted for age, gender and hypertension. Open circles connected by the dashed line represent the reference value (1) for each OR. Odds Ratios are represented by solid circles and error bars, which represent 95% confidence intervals. The percentage of psoriasis patients (**a**), psoriatic arthritis patients (**b**) or control subjects (**c**) exhibiting either myocardial infarction (MI), atrial fibrillation (AF) and chronic heart failure in psoriasis patients based on MPV expression levels is shown. Prediction of odds ratio for development of the three types of heart failure in psoriasis (**a**) psoriatic arthritis (**b**) and controls (**c**) are quantified on the right axis.

Among patients with PsO, those with increased MPV also had an increased odds ratio of cardiovascular events compared to PsO patients with normal/low MPV: MI of 1.52 (95%CI 1.3–1.78); AF of 1.71 (95%CI 1.47–1.99); and heart failure of 2.34 (95%CI 2.03–2.71) (Figure 2a). In patients with PsA and elevated MPV (*n* = 1850 (8.33%), relative to PsA with normal/low MPV there was an increased odds ratio for AF of 1.69 (95%CI 1.27–2.24), and CHF 2.24 (95%CI 1.71–2.95), but not for MI (OR 1.0, 95%CI 0.74–1.35) (Figure 2b). Increased MPV among control patients increased odds of MI by 1.63 (95%CI 1.6–1.65), AF by 1.66 (95%CI 1.63–1.69), and CHF by 2.53 (95%CI 2.5–2.57); values comparable to published reports (Figure 2c) [10].

In comparison to healthy control subjects, patients with PsO (without psoriatic arthritis), and high MPV had increased odds of MI (OR 1.44 95%CI 1.25–1.66), AF (OR 1.79, 95%CI 1.57–2.05), and CHF (OR 1.46, 95%CI 1.29–1.65) (Supplementary Figure S3). In addition, PsA patients with high MPV also had higher odds of AF (OR 1.52, 95%CI 1.17–1.97), but not MI (OR 1.17, 95%CI 0.88–1.55) or CHF (OR 1.23 95%CI 0.97–1.56).

### 3.4. Combination of MPV and RDW in Assessment of Cardiovascular Disease Risk

**Figure 3:**
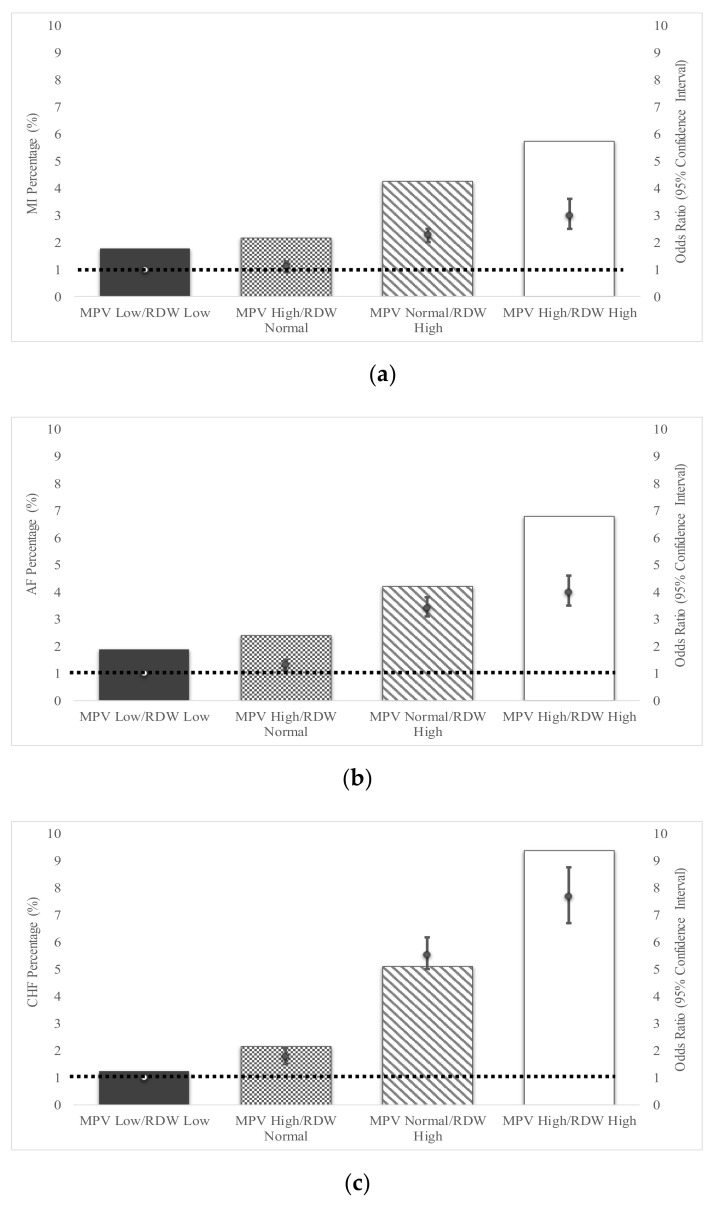
Percentage of Psoriasis patients exhibiting Cardiovascular Disease based on a combination of Mean platelet volume (MPV) and Red Cell Distribution Width (RDW). The percentage of cardiovascular disease; myocardial infarction (MI), (**a**), atrial fibrillation (AF), (**b**), and chronic heart failure (CHF), (**c**) based on a combination of red cell distribution width and mean platelet volume in psoriasis patients is shown along with the Odds Ratio of developing these conditions as predicted by the combined values of RDW and MPV. Odds Ratios are adjusted for age, gender and hypertension. Open circles connected by the dashed line represent the reference value (1) for each OR. Odds Ratios are represented by solid circles and error bars, which represent 95% confidence intervals.

Data for both MPV and RDW PsO and PsA patients was available for 89,710 patients. Elevated RDW and MPV was present in 3990 (4.45%) psoriasis patients, elevated RDW and normal MPV was present in 12,100 (13.5%), while 8120 (9.05%) patients had normal RDW and elevated MPV. After adjusting for age, gender and hypertension, prevalence of MI was highest among psoriasis patients who had both high RDW and MPV (5.3%, OR 3.0, 95%CI 2.56–3.49), followed by high RDW and normal MPV (3.9%, OR 2.3, 95%CI 2.09–2.6), normal RDW with high MPV (1.97%, OR 1.1, 95%CI 0.95–1.33) and lastly low MPV and low RDW (1.9%, Figure 3a). Similarly, prevalence of AF was highest among those psoriasis patients with high RDW and MPV (5.26%, OR 3.97, 95%CI 3.44–4.57), followed by high RDW and normal MPV (3.9%, OR 3.4, 95%CI 3.1–3.8), normal RDW with high MPV (1.97%, OR 1.3, 95%CI 1.1–1.53) and lastly low MPV and low RDW (1.89%, Figure 3b). Prevalence of CHF among psoriasis patients with both high RDW and MPV (8.4%, OR 7.7, 95%CI 6.7–8.8), was highest, followed by high RDW and normal MPV (5.95%, OR 5.6, 95%CI 5.0–6.2), normal RDW with high MPV (1.97%, OR 1.8, 95%CI 1.5–2.1) and lastly low MPV and low RDW (1.2%, Figure 3c). The low MPV and low RDW groups are the reference groups when calculating the ORs.

### 3.5. Responsiveness of High RDW to Therapy

**Figure 4:**
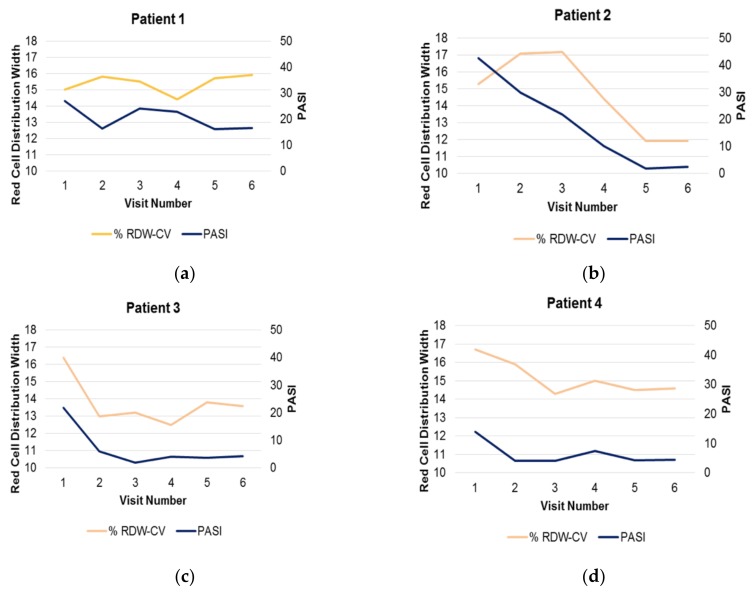
Alteration in red cell distribution (RDW) and psoriasis area and severity index (PASI) following therapeutic treatments. Change in RDW and PASI are indicated on the left and right axes, respectively. Patients were treated for the indicated time with several independent systemic therapies including acitretin and phototherapy (**a**), methotrexate followed by adalimumab (**b**), infliximab and methotrexate (**c**) and an additional methotrexate followed by adalimumab (**d**).

In a dataset of 24 patients who were followed for 1 year while receiving systemic therapy, 4 patients (16.7%) had elevated RDW at baseline which was similar to the 21% high RDW figure in the Explorys group. Patients 2, 3, and 4 were responders to therapy (each achieved PASI <5) and their RDW normalized after therapy (Figure 4). The PASI’s were correlated with their RDW (patient 2: *r* = 0.75, patient 3: *r* = 0.92 and patient 4: *r* = 0.77; Figure 4b–d). In contrast, patient 1 who was a non-responder, exhibited minimal change in their PASI (from 23 to 17), and the RDW actually increased during this time (Figure 4a). RDW thus may identify a dynamic, endotypic biomarker response of psoriasis patients in whom skin inflammation results in a distant organ (bone marrow) response. In the larger group of 24 which included data-points over a one year period on both normal and high RDW patients, RDW was not correlated with raw PASI (*r* = 0.27), suggesting that only a subset of patients with psoriasis have a type of inflammation capable of modifying the RDW.

### 3.6. Regional Organ Response Compared to Distant Organ Response

**Figure 5:**
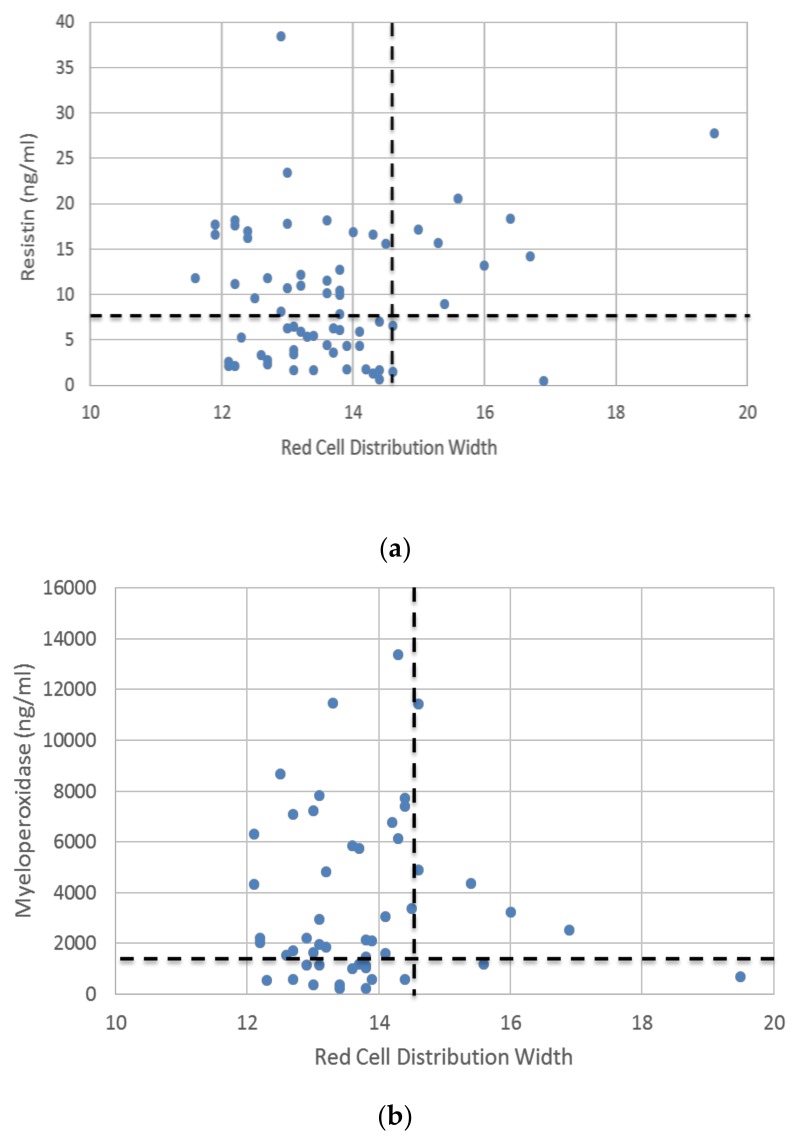
Elevated serum resistin in conjunction with elevated RDW marks a subset of psoriasis patients experiencing distal organ response. Among 75 patients recruited in our Murdough Family Center for Psoriasis that had RDW and resistin levels recorded, nearly half of the psoriasis patients had elevated resistin, and nine patients had elevated RDW, which represented 24% of the resistin high subset (**a**). Of the nine patients with high RDW, eight (88%) demonstrated elevated resistin. In contrast, elevated resistin was present in only 26 (39%) patients with normal RDW. Among 51 patients with MPO and RDW observed, 73% had elevated MPO levels and 8 patients had elevated RDW values which represented 14% of the MPO high subset (**b**). Among the high RDW patients, six (75%) also had elevated MPO values.

In a dataset of 75 patients in whom RDW and resistin levels were obtained, almost half of the psoriasis patients had elevated resistin, and nine patients had elevated RDW, which represented 24% of the resistin high subset (Figure 5a). Of the nine patients with high RDW, 8 (88%) demonstrated elevated resistin. By contrast, elevated resistin was present in only 26 (39%) patients with normal RDW. Among the 51 patients with recorded MPO and RDW values, 73% had elevated MPO levels and 8 patients had elevated RDW values which represented 14% of the MPO high subset (Figure 5b). Among these high RDW patients, 6 (75%) had elevated MPO values. Thus, resistin, which is elevated in a minority of patients with moderate-severe psoriasis, may be more linked to mechanisms associated with RDW elevation than MPO, which may simply reflect skin release.

## 4. Discussion

Recent publications have demonstrated that elevated MPV and RDW may be used as predictors for increased CVD risk [40]. In this analysis, we examined MPV and RDW separately and in combination in patients with psoriatic disease. In particular, the highest CVD risk was seen in the patients with a combination of elevated MPV and RDW, in which psoriasis patients with high values of both had more than 3-fold risk of MI and >7 fold risk of AF or CHF than psoriasis patients with normal/low values of both RDW and MPV. Our data suggest that MPV and RDW may be better predictors of MI in psoriatic than in PsA patients, potentially because cutaneous inflammation identified by pro-inflammatory cytokines is more easily distributed compared to cytokines derived from joints due to their anatomical differences.

We also observed in a limited number of patients with elevated RDW who were longitudinally followed over a year, that effective psoriasis therapy correlated with a decrease or normalization of RDW values. This suggests that RDW can be a relatively dynamic biomarker of distant organ responses to psoriatic inflammation. Finally, when we examined the adipocyte-derived resistin biomarker, we found a high percent of patients with high RDW that also had high resistin values, perhaps signifying that adipocyte inflammation may mark a subtype of psoriatic inflammation capable of triggering systemic as well as sub-adjacent organ inflammation.

We demonstrated that there are progressive changes in the degree of extra-cutaneous impact among patients with psoriasis. For example MPO is elevated in 73% of patients, likely reflecting vascular uptake from areas in the dermis where high concentrations of MPO are released by myeloid cells [42]. In addition, mediators from the dermis reaching the vasculature may be triggering PMN-derived and monocyte-derived MPO release [43]. Resistin is elevated in 52% of patients, a smaller subset than that of MPO, likely reflecting psoriatic dermal-epidermal mediators activating the underlying adipose tissue [44], although myeloid release is also possible. However, a much smaller subset, 4.45% of the patients, appears to demonstrate both high RDW and high MPV and assuming that both RDW and MPV are biomarkers of cardiovascular risk and damage, these patients may display a larger cardiovascular impairment. Thus, critically regarding the identified subsets (endotypes) based on the levels of two well-known cardiovascular biomarkers, RDW and MPV, it is expected that each of these subsets will have a different CV risk and consequently a different risk to develop CV-related comorbidities.

RDW is perturbed by microvascular, bone marrow, and neurohumoral/neuroendocrine dysfunction. Variation in size of RBCs is a result of bone marrow modulation and disordered production of RBCs [45]. Microvascular abnormalities related to endothelium activation, such as in psoriasis or CVD can result in endothelial cytokine release including IL-6 that can circulate into the marrow and affect erythropoiesis [46]. Bone marrow alterations affecting erythrocytes can result in increased stimulation of erythrocyte progenitors as well as decreased erythropoietin-induced differentiation of erythrocytes [47]. Signaling through neuroendocrine and hypothalamic pituitary-adrenal axes can also modify bone marrow stem cells and erythrocyte stimulation and release [48]. Specifically, psoriasis-related pro-inflammatory cytokines may influence all these aspects of the bone marrow and neurohumoral/neuroendocrine function, giving a rationale for the observation of increased RDW found in psoriasis patients.

MPV is a parameter that reflects platelet activation, bone marrow function and microvascular dysfunction [49]. MPV correlates with RDW, and together they may inform the complexity of pro-inflammatory status associated with psoriasis from two different perspectives, namely RDW from the erythrocyte lineage and MPV from the platelet lineage. Both platelet and erythrocyte alteration have been reported to be involved in cardiovascular risk and psoriasis [50].

RDW, in combination with MPV, may also be interpreted as monitors of bone marrow health. This is demonstrated by increased RDW with age as well as increased rates of hematologic malignancies with higher MPV and RDW which are considered negative independent prognostic factors of survival in myelofibrosis and myelodysplasia [45,51]. In fact Sousa et al., advocated RDW as a novel marker of stress erythropoiesis, which may be induced by systemic inflammatory conditions such as psoriasis [52]. Several abnormalities in bone marrow stem cells of psoriatic patients have been previously described, due to a modification of bone marrow microenvironment based on a psoriasis-related pro-inflammatory skin [17]. Pro-inflammatory cytokines, microvascular disorder, and dysregulated erythropoiesis contribute to increased cardiovascular risk. In accordance with recent publications, RDW is a reliable, strong prognostic marker of cardiovascular disease, and MPV contributes to a more detailed assessment of cardiovascular risk in patients that have a lower level of inflammation [16,47].

The endocrine aspects of our study were further investigated by assessing the relationship between resistin and RDW. Resistin is primarily produced by adipocytes, as well as macrophages, and neutrophils. Resistin levels are increased in several autoimmune conditions, and plays a role in insulin resistance and in cardiovascular remodeling via inhibition of AMP-activated protein kinase (AMPK) [23,53,54,55,56]. Pro-inflammatory neutrophil activation is also enhanced and promoted by resistin.

Prior studies have identified the increased risk for CVD in psoriasis patients [57] but few have practical ways for the dermatologist to identify patients at increased risk [58]. In cardiology, identification of patients at higher risk are focused on (1) increasing accuracy of algorithms such as Framingham Score and the ACC/AHA pooled cohort equation and (2) identifying biomarkers [58]. Remarkably, the ACC/AHA pooled cohort equation may under-represent CV risk, or is understudied in the PsO/PsA population, therefore validating that easy-to-use routine biomarkers would be clinically useful [59].

This study also has limitations. In the Explorys extracted cohort, we are unable to adjust for lifestyles, prescriptions, and severity, which may potentially be effect modifiers. On the other hand, the study’s large geographic distribution and population data make the conclusions more generalizable, which we were able to confirm in our small institutional cohort and include longitudinal data. An important weakness of the study is that we were able to analyze only a point-in-time determination of the parameters under study (RDW/MPV), although it is well established that both are modulatory parameters that can be influenced by multiple conditions. Importantly, the correlation with resistin, myeloperoxidase, and the effect of psoriasis treatment on the studied parameters is preliminary and was performed on a significantly smaller cohort than the other analyses. Further studies are needed in order to define psoriasis endotypes and address personalized treatment based on these endotypes.

## 5. Conclusions

The findings presented here indicate that a simple, inexpensive complete blood count (CBC) can identify a subset of psoriasis and psoriatic arthritis patients who should receive close clinical attention for cardiovascular event occurrence and consideration of preventive interventions.

The detection of subgroups with increased CVD risk markers and subgroups with less evidence of systemic inflammation, highlights the concept of endotypes-clinical and laboratory characteristics which define the biological signature of comorbidities in the single patient.

However, using RDW or MPV to assess CV prognosis remains investigational, and how best to interpret it in daily practice requires further study. RDW can be abnormal in anemia and needs to be evaluated independently of the psoriasis. Like the RDW, MPV must be interpreted in conjunction with the platelet count, and an abnormal value should always be confirmed with evaluation of a peripheral blood smear. The RDW and MPV count will not likely provide definitive or flawless diagnostic or prognostic information, but when understood and used correctly, they may provide readily available, cost-effective, and useful data that can supplement and guide clinical decision-making.

## Figures and Tables

**Table 1:** Clinical and demographic characteristics of the cohort downloaded from Explorys database (Run date 07/09/2018).

	Psoriasis (*n* = 91,190 (%))	Psoriatic Arthritis (*n* = 22,220 (%))	Control (*n* = 10,633,910 (%))
**Age (years)**			
<40	23,010 (25.23)	3320 (14.94)	4,801,560 (45.15)
40–49	21,270 (23.32)	5420 (24.39)	2,275,540 (21.4)
50–59	28,750 (31.53)	8520 (38.34)	2,395,360 (22.53)
60–65	18,160 (19.91)	4960 (22.32)	1,161,450 (10.92)
**Gender**			
Male	43,720 (47.94)	11,550 (51.98)	4,360,730 (41.01)
Female	47,470 (52.06)	10,670 (48.02)	6,271,350 (58.98)
Not Recorded	0 (0)	0 (0)	1830 (0.02)
**Race**			
Caucasian	76,940 (84.37)	17,180 (77.32)	7,405,980 (69.64)
African-American	5370 (5.89)	1480 (6.66)	1,481,020 (13.93)
Other	8880 (9.74)	3560 (16.02)	1,746,910 (16.43)
**BMI (kg/m^2^)**			
Underweight (<18.5)	3540 (3.88)	500 (2.25)	731,070 (6.87)
Normal (18.5–24.99)	12,610 (13.83)	2310 (10.4)	3,079,670 (28.96)
Overweight (25–29.99)	23,570 (25.85)	4840 (21.78)	2,412,230 (22.68)
Obese (>30)	28,200 (30.92)	10,070 (45.32)	3,567,680 (33.55)
Not Recorded	23,270 (25.52)	4500 (20.25)	843,260 (7.93)
**Smokers**	24,720 (27.11)	4560 (20.52)	1,634,230 (15.37)
**Blood pressure (mmHg)**			
Normal (<120 systolic, <80 diastolic)	4770 (5.23)	4530 (20.39)	1,169,620 (11)
Prehypertension (120–139 systolic, 80–89 diastolic)	25,360 (27.81)	4890 (22.01)	3,144,250 (29.57)
Stage 1 Hypertension (140–159 systolic, 90–99 diastolic)	30,300 (33.23)	6680 (30.06)	2,540,710 (23.89)
Stage 2 Hypertension (>160 systolic, >100 diastolic	23,780 (26.08)	5190 (23.36)	1,548,290 (14.56)
Not Recorded	6980 (7.65)	910 (4.1)	2,231,040 (20.98)
**RDW (%)**			
High (>14.5)	19,480 (21.36)	5210 (23.45)	1,943,130 (18.27)
Normal/Low (≤14.5)	69,670 (76.4)	16,660 (74.98)	8,311,460 (78.16)
No RDW Data Available	2040 (2.24)	350 (1.58)	379,320 (3.57)
**MPV (fL)**			
High (>12.3)	7900 (8.66)	1850 (8.33)	747,140 (7.03)
Normal/low (≤12.3)	64,180 (70.38)	17,780 (80)	5,921,370 (55.68)
No MPV Data Available	7110 (21.02)	2590 (11.68)	9,965,400 (37.29)

BMI: body mass index, MPV: mean platelet volume, RDW: red cell distribution width.

## Supplementary Materials

The following are available online at https://www.mdpi.com/2077-0383/9/1/186/s1, Figure S1: Inclusion and exclusion criteria for selection of psoriasis patients from the Explorys database, Figure S2: Predicted Odds Ratio of developing Cardiovascular Disease based on high versus normal Red Cell Distribution Width (RDW) for Psoriasis patients versus Psoriatic Arthritis patients versus Controls, Figure S3: Predicted Odds Ratio of developing Cardiovascular Disease based on high versus normal Mean Platelet Value (MPV) for Psoriasis patients versus Psoriatic Arthritis patients versus Controls.
