# Supplementary material for: Psoriasis and Psoriatic Arthritis Cardiovascular Disease Endotypes Identified by Red Blood Cell Distribution Width and Mean Platelet Volume
Source: J Clin Med. 2020 Jan 9;9(1):186. doi: 10.3390/jcm9010186 (PMC7019311; doi:10.3390/jcm9010186)
Supplement: Supplementary file 1 [file jcm-09-00186-s001.pdf]

Supplementary Figure S1. Inclusion and exclusion criteria for selection of psoriasis patients from the Explorys database

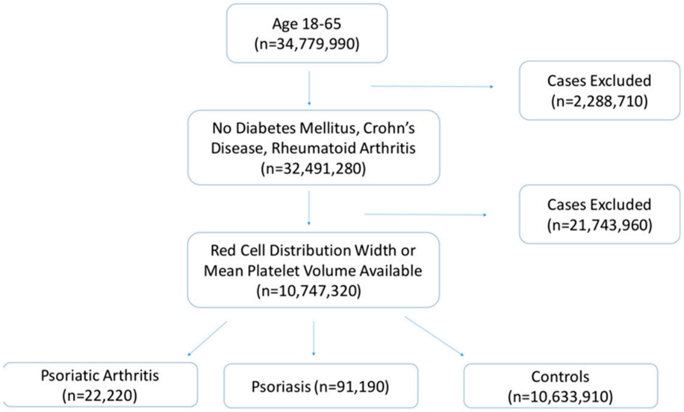

The aggregate patient database Explorys contains data from approximately 55 million patients. We selected from enrolled subjects with an age range of 18-65 and excluded subjects with a diagnosis code indicating Diabetes Mellitus, Crohn's or Rheumatoid Arthritis (n = 32,491,280). Of these subjects, those with available data on Red Cell Distribution Width (RDW) or Mean Platelet Volume (MPV) were identified (n = 10,747,320). Among these patients, we then identified subjects with either psoriatic arthritis (PsA, 22,220), psoriasis (PsO, 91,190) or those subjects not diagnosed for either PsA or PsO (10,633,910) as controls.

Supplementary Figure S2. Predicted Odds Ratio of developing Cardiovascular Disease based on high versus normal Red Cell Distribution Width (RDW) for Psoriasis patients versus Psoriatic Arthritis patients versus Controls.

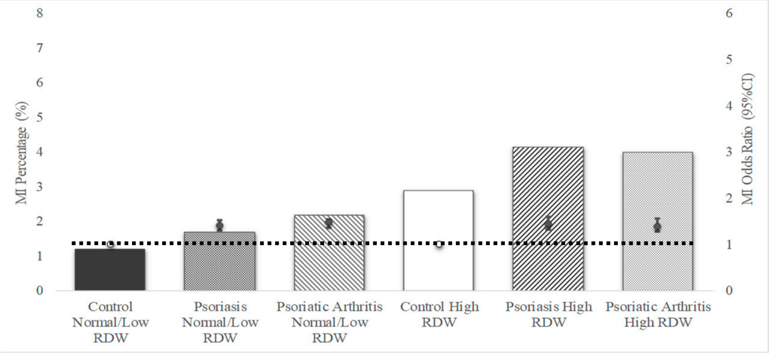

a)

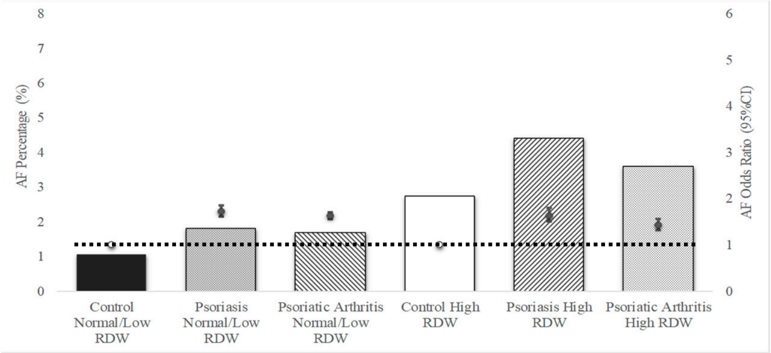

b)

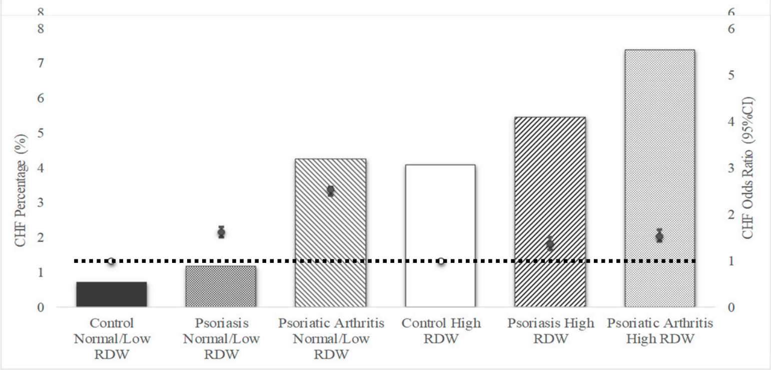

c)

The odds ratio of presenting with three distinct types of cardiovascular diseases based upon RDW status (high versus normal/low, as defined by Explorys) is shown for Psoriasis and Psoriatic Arthritis patients versus Controls (as defined in Figure 1). The percentage of patients (PsO, PsA) or controls exhibiting each combination is demarcated on the left hand side of the graph, while the odds ratios (OR) scale is listed on the right hand side. Odds Ratios were adjusted for age, gender and

hypertension. Open circles connected by the dashed line represent the reference value (1) for each OR. Error bars represent 95% confidence intervals. The percentage of Myocardial Infarction (MI, S2a), Arial Fibrillation (AF, S2b) and Chronic Heart Failure (S2c) among psoriasis patients, psoriatic arthritis patients or control subjects based on RDW expression levels is shown. Prediction of odds ratio for development of the three types of heart failure in psoriasis, psoriatic arthritis and controls subjects based on RDW levels are quantified on the right axis.

Supplementary Figure S3. Predicted Odds Ratio of developing Cardiovascular Disease based on high versus normal Mean Platelet Value (MPV) for Psoriasis patients versus Psoriatic Arthritis patients versus Controls.

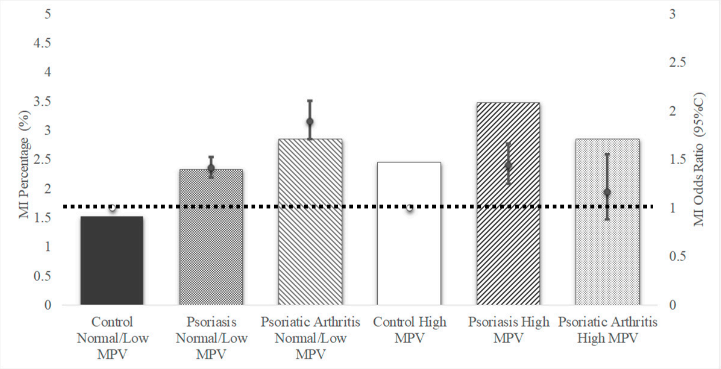

a)

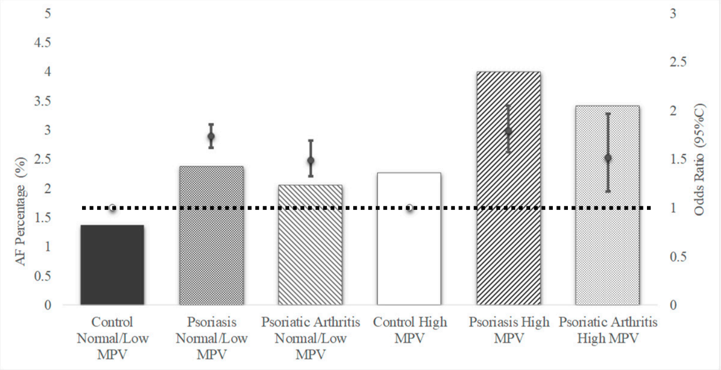

b)

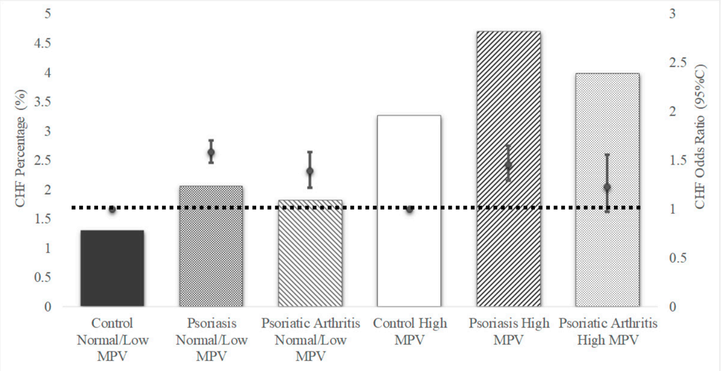

c)

The odds ratio of presenting with three distinct types of cardiovascular diseases based upon MPV status (high versus normal/low, as defined by Explorys) is shown for Psoriasis and Psoriatic Arthritis patients versus Controls. The percentage of patients (PsO, PsA) or controls exhibiting each combination is demarcated on the left hand side of the graph, while the odds ratios (OR) scale is listed on the right hand side. Odds Ratios were adjusted for age, gender and hypertension. Open circles

connected by the dashed line represent the reference value (1) for each OR. Error bars represent 95% confidence intervals. The percentage of Myocardial Infarction (MI, S3a), Arial Fibrillation (AF, S3b) and Chronic Heart Failure (S3c) among psoriasis patients, psoriatic arthritis patients or control subjects based on MPV expression levels is shown. Prediction of odds ratio for development of the three types of heart failure in psoriasis, psoriatic arthritis and controls subjects based on MPV levels are quantified on the right axis.
